# Barriers and facilitators in implementing advance care planning for frail older patients acutely admitted to geriatric hospital units: a nested qualitative study

**DOI:** 10.3389/frhs.2025.1646541

**Published:** 2025-12-17

**Authors:** Linn Brøderud, Maria Romøren, Karin Berg Hermansen, Trygve Johannes Lereim Sævareid, Lisbeth Thoresen, Reidar Pedersen

**Affiliations:** 1 Centre for Medical Ethics, Institute of Health and Society, Faculty of Medicine, University of Oslo, Oslo, Norway; 2 Department of General Practice, Institute of Health and Society, Faculty of Medicine, University of Oslo, Oslo, Norway; 3 Department of Health Sciences, Aalesund, Faculty of Medicine and Health Sciences, Norwegian University of Science and Technology, Aalesund, Norway; 4 Department of Health and Nursing Science, University of Agder, Kristiansand, Norway; 5 Department of Public Health and Interdisciplinary Health Sciences, Institute of Health and Society, University of Oslo, Oslo, Norway

**Keywords:** advance care planning, communication, complex interventions, frailty, geriatrics, implementation, hospital, older adults

## Abstract

**Background:**

Appropriate communication with patients is increasingly crucial in a growing elderly population to prevent both over- and undertreatment. Advance care planning (ACP) is recognized as a valuable communication process for patients, their relatives and healthcare professionals that facilitates future care and medical decision-making. Despite its importance, the uptake remains low, particularly among frail, older patients in hospitals.

**Methods:**

This qualitative substudy is nested within a cluster randomized controlled trial. Data collection involved eight semi-structured interviews conducted in acute geriatric hospital units receiving our implementation support program, along with informal data from interactions with the units during the implementation process. The aim was to explore healthcare professionals' perspectives on the barriers and facilitators to ACP implementation and their experiences with the implementation support program. A semi-structured interview guide was used. The data was analyzed using content analysis.

**Results:**

Factors influencing ACP implementation were identified at three levels: a) the organizational level, b) the national level, and c) the clinical level. Participants recognized the critical role of timing, context, and patients' capacity. However, there was meaningful opportunities for ACP conversations in acute geriatric units. Overall, the experiences underscored the complex interplay of individual motivation and interest, organizational support, prioritization, available time and resources, and systemic factors that influence the integration of ACP into clinical practice, as well as the fact that research can act as both a barrier and a facilitator in implementation efforts.

**Discussion:**

This study illustrates the significant challenges in implementing ACP in acute hospital care. Despite a generally positive perception of ACP, its implementation was hindered by barriers such as overwhelming workload, production-oriented healthcare, the biomedical model, and lack of prioritization. These factors creates a cycle where short-term demands overshadow preventive and patient-centered interventions, limiting their perceived and documented benefits. Breaking this cycle will likely require targeted investment in the implementation of complex interventions.

**Trial registration:**

ClinicalTrials.gov, Identifier NCT05681585.

## Introduction

The aging population has significant implications for the planning and delivery of health and social care (1, 2). In line with the world's growing population, more people will experience cognitive impairment, frailty and comorbidity which are associated with higher rates of adverse health outcomes (3). Frailty is associated with higher levels of disability, falls, delirium and death, use of hospital services, unpredictable dying trajectory (1, 4, 5) and higher healthcare costs (6). Making treatment decisions for frail patients is a challenge due to the complex interplay of their multiple comorbidities and the delicate balance of risks vs. benefits. It becomes even more difficult when older patients are no longer able to express their preferences (7). In addition, research suggests that relatives or healthcare professionals (HCPs) may not know or predict patients' health preferences (8), and it is known that frail older people and their relatives are often inadequately involved in decision-making (9, 10). Increasing medical technologies and innovations offer the opportunity to prolong an individual's life. However, this may be at the expense of quality of life (11, 12) or other needs, and ethical challenges arise (13), such as overtreatment and potentially unnecessary and unwanted interventions (7, 14). Respecting patient autonomy in decision-making is a fundamental ethical principle (7). Considering these issues in conjunction with resource utilization (15), it is imperative to set good priorities, both to avoid interventions that patients may not want and to ensure that patients are neither under- nor overtreated. With better communication, documentation and planning, many negative outcomes could be avoided (16–18). Therefore, advance care planning (ACP) is becoming increasingly important (7, 17, 19).

ACP was previously defined by two consensus studies (20, 21). ACP is now recognized as a communication process for patients, their relatives and HCPs that facilitates future care and medical decision making in accordance with the patient's values, preferences and physiological, social and spiritual concerns (21–24). A recent systematic review highlights that ACP can improve outcomes such as quality of patient-physician communication, preference for comfort care, reduction in decisional conflict, and greater patient-caregiver congruence in preferences, as well as improved documentation (25).

### Knowledge gap, context and relevance

Only recently, the first national ACP guideline was published in Norway (26). While many countries have enshrined ACP in law (27), Norway has no such legislation. However, ACP has been recommended in broader Norwegian guidelines and policies for over a decade (13, 28–30), but uptake and dissemination has been limited (31). There is evidence that the introduction of ACP in nursing homes may come too late (32–34), but there is still a lack of evidence on when, where and who should introduce ACP in order to mainstream it more widely into routine care (35). Hospitalization may provide an opportunity for ACP given the high use of hospital services by frail older patients (4, 19, 36). It often represents an acute deterioration in health, life-sustaining interventions are often initiated here (37) and the patient is inevitably confronted with their health situation, values and decisions to be made (38). Furthermore, older people may prefer to talk sooner rather than later (39), and a smaller study found that ACP was well received by geriatric patients during their hospitalization (40). A systematic review found that the rate of ACP in hospitalized older patients is low, and research on the perceptions of HCPs in hospitals is sparse, particularly among physicians (36).

There is a large body of research on the potential benefits of ACP (3, 25, 36, 41–43), the overall positive attitudes and global effort to implement it (43), but uptake remains generally low (3, 7, 15, 31, 36–38, 43–45). Although ACP is particularly relevant for frail older people (36, 46), most studies of ACP have focused on patients with specific conditions, living in nursing homes or in palliative care settings. Few have focused on frail geriatric patients admitted acutely to hospital (14, 15, 38, 43, 47). Furthermore, there are no clear, generally accepted guidelines for the implementation of ACP (43). It is possible that insufficient attention has been given to the complexity of ACP implementation (48–50). The ACP process often involves multiple professionals and multiple steps, targeting patients with different diseases and disease trajectories and their relatives. ACP is conducted and followed up across complex healthcare systems and communities with different workflows, policies and societal norms (24). Therefore, ACP can be defined as a complex intervention (46, 48–50). It involves preparing and planning for an uncertain future that requires nuance and a balance between hope and reality (51). Complexity also arises from the conceptualization of ACP as a whole process that involves numerous interconnected elements and stakeholders (43). However, there are few studies that address the implementation of ACP as a complex intervention (48–50, 52). Implementation science can provide frameworks and tools to better understand the complex processes involved in implementing complex interventions and the reasons for implementation success or failure (53, 54). This study was guided by the following research question: “What barriers and facilitators do local implementation teams and regular clinicians in acute geriatric units experience when implementing ACP into routine practice for frail older patients following an implementation support program (ISP)?”.

## Methods

### Study design

This qualitative study is nested within the complex cluster randomized controlled trial (cRCT) “Implementation of advance care planning in the routine care for acutely admitted patients in geriatric units” (35). The cRCT comprised a clinical intervention, ACP, and an implementation intervention, namely the implementation support program (ISP). Twelve hospital units were randomly assigned to either the intervention group (receiving the ISP) or the control group (care as usual). This qualitative nested study is conducted within the six intervention units. The project as a whole (35) was based on both responsive (55) and formative (56) evaluation approaches. This was operationalized through continuously discussions about the key barriers and facilitators with the participants, namely the clinicians in the implementation teams in the intervention units (35). This feedback was incorporated into both the ISP and the clinical intervention.

### The clinical intervention, ACP

The clinical intervention is based on previous research conducted by the Center for Medical Ethics, including topics such as end-of-life ethics, patient participation, decision-making for older patients and their relatives, ACP in nursing homes, and national and international research and guidelines on ACP and end-of-life decision making. The clinical intervention consisted of the following: (a) routine identification, information and invitation to ACP to all eligible patients and their relatives, (b) ACP conversations routinely provided to all consenting patients and their relatives, and (c) documentation and collaboration with other health care services and levels (35). The cRCT had an approach to ACP that encouraged the involvement of relatives. The project also allowed ACP conversations with only the relatives when patients were unable or chose not to participate.

### The implementation support program

The developed ISP (35) was inspired by previous implementation projects of the Center for Medical Ethics (57, 58) and implementation science. The ISP originally consisted of the implementation strategies and measures listed in Table 1. Intervention units received implementation support for 26 months (October 2022–December 2024) to integrate ACP into clinical practice. We emphasized a whole ward approach aimed at engaging all relevant HCPs in the units and not just individuals (59), as well as a “train the trainer” model in which dedicated staff were equipped with the necessary skills to train colleagues in local context (60).

**Table 1:** Overview of the implementation strategies and measures.

**Implementation strategies:** Whole ward approach. Train the trainer model. Promote adaptability through responsive evaluation. Formative evaluation. Leadership commitment. Sustainability after project. **Implementation measures:** Identify and prepare champions. A startup meeting on implementation effort for all relevant HCPs in the unit. The implementation team develops an action plan with assistance from the research group. Revise the action plan in light of feedback and supervision after fidelity measurements. Local implementation team comprising of 1–5 clinicians and preferably a leader. Appoint an ACP coordinator. Develop a reminder system to aid the use of ACP. Routine and workflow identification of ACP, as well as documentation routines of ACP. Training, education and supervision in both implementation intervention and clinical intervention: Kick-off, training of resource persons and HCPs including education in ACP, emphatic clinical communication and ACP simulation, overview of barriers and potential solutions to ACP, and ethical and legal aspects of ACP. Freely available ACP toolkit and resources: ACP guideline, pocket card with conversation topics prompts and key points to consider before, during and after ACP, teaching materials such as PowerPoints and videos, information leaflets, and documentation templates for journaling and an example of how to document an ACP. Regular dialogue meetings with units and annual network meetings to collect local needs and sharing experiences. Available overview of known barriers and potential solutions (facilitators). Structured fidelity measurements to monitor the current level of implementation at baseline, at 7–10 months and 23–25 months after baseline. Tailored feedback and supervision at each unit after each fidelity measurement, using the preliminary results from the fidelity scale: a) implementation level, b) quality of ACP and c) penetration rate. Implement a systematically organized evaluation of both ACP and the implementation strategies including inputs from clinicians, patients and their relatives. Preliminary results were used to adapt the clinical and implementation intervention. Assess and possibly adjust which professionals should be responsible or participate in ACP. Tailor the implementation strategies to address barriers and facilitators. Seek input and guidance from experts and users outside the implementation team and project group.

As a core part of the ISP, each unit was recommended to appoint a local coordinator for ACP to ensure that ACP took place. An implementation team was also recommended, consisting of HCPs and preferably a manager to be responsible for local implementation and act as a bridge between the research group and the unit. To promote adaptability, the research group collaborated with the units to create ACP materials and resources. The ISP began with a kick-off day for the six acute geriatric units, where the implementation teams were informed about the project and educated on ACP. This was followed by a two-hour ACP training session for all clinical staff in each unit at the baseline of the project. The implementation teams received a full day of hands-on training on ACP led by a phycologist specialized in clinical communication. The implementation teams were encouraged to expand the application in the local context through the train-the-trainer model.

### Context and study setting

We decided to investigate the implementation of ACP in acute geriatric hospital units, as they have expertise in interdisciplinary, patient-centered and holistic care of frail older patients, who often have multiple diseases and a limited life expectancy. 12 out of 14 eligible units in hospitals in the South-Easters Norway Regional Health Authority agreed to participate in the cRCT. The units included in this nested study were all classified as acute geriatric units except for one that was classified as an internal medical unit with at least one geriatrician among staff. These units treat patients from a catchment area corresponding to 43% of the total Norwegian population of 5.5 million people. Those that declined to participate cited insufficient capacity to participate in research projects.

The organization and size of the included units varied between the included hospitals (see Table 2). All units were responsible for the hospital care of acutely admitted ill, home-dwelling and frail older patients who may benefit from a multidisciplinary geriatric team approach. In this approach, functional and cognitive abilities and degree of frailty are assessed to create an individualized plan that focuses on maintaining overall functioning and treat acute and chronic illness (35).

**Table 2:** Description of the acute geriatric units.

Site	Type of unit^a^	Catchment population	Number of geriatric beds	Mean length of stay for acute geriatric patients (days)	Full-time equivalent staff (FTE)	Patients per FTE
1	IMU	50,000	6	4.55	23	3.83
2	AGU	120,000	9	4	40	4.44
3	AGU	155,000	4	2.5	19.8	4.95
4	AGU	230,000	9	4	16.5	1.83
5	AGU	323,453	18	4	47.2	2.6
6	AGU	594,000	23	6	53.5	2.32

a AGU, acute geriatric unit; IMU, internal medical unit with geriatrician among staff.

### Data collections

This qualitative study explored the experiences of HCPs during the implementation period using formal and informal data collection methods. This included eight semi-structured interviews, feedback collected during fidelity assessments, and insights from regular dialogue and network meetings, with written notes from these meetings forming part of the data. The semi-structured interviews were conducted in all six intervention units, including four focus groups (three to five participants) and four interviews with two participants, involving a total of twenty-four clinicians. Two of the participating clinicians also held managerial roles and responsibilities in the participating units. The characteristics of the participants are summarized in Table 3. Participants were contacted by email and received information, topic notes and a consent form. The interviews took place between December 2023 and October 2024 at the participants' workplaces. The interviews lasted one to one and a half hours each. The interviews were digitally recorded and transcribed verbatim by LB and KBH. The most important impressions from the interviews were then subsequently shared with the research group.

**Table 3:** Overview of characteristics of the participants.

Characteristics	Members of local implementation teams (*n* = 12)	Regular clinicians (*n* = 12)
Sex		
Male	2	4
Female	10	8
Age in years		
25–34	3	4
35–44	3	7
45–54	3	1
55–64	3	0
Professional background		
Specialist in internal medicine and geriatrics	6	1
Specialist in internal medicine	1	2
Medical doctor in specialization	0	4
Nurse	4	5
Other	1	0
Years of experience in total		
<1		
1–3	1	3
4–9	2	4
10–15	1	3
>15	8	2
Years at the acute geriatric unit		
<1		3
1–3	1	5
4–9	9	4
10–15	1	0
>15	1	0

The semi-structured interview guide was inspired by previous implementation research projects and other relevant research conducted by the Center for Medical Ethics (57, 59) and further refined by the research group. It addressed all domains of the Consolidated Framework for Implementation Research (CFIR): the “inner setting domain” (the healthcare organization and unit in which the intervention or innovation is implemented), the “individual domain” (the roles and characteristics of individuals), the “implementation process domain” (the activities and strategies used to implement the clinical intervention), the “innovation domain” (the “thing” being implemented, which in this study is ACP), and the “outer setting domain” (the context in which the inner setting exists) (61). The latter domain is explored in more detail in another substudy (62). The interview guide was pilot tested and slightly modified (See Supplementary File 1 for the interview guide).

### Research team and reflexivity

All interviews were conducted by two researchers together: LB (RN, MScN, PhD Fellow) in six interviews, KBH (ICU nurse, PhD Fellow) in six interviews, MR (Assoc. Prof. and medical doctor) and TJLS (Assoc. Prof. and RN) both in two interviews. A relationship already existed between most of the interviewees and the interviewers due to the ISP. LB and KBH were the least experienced qualitative researchers, while MR, TJLS, RP (Prof. of medical ethics, BA, MA and medical doctor) and LT (Assoc. Prof. and RN) are well experienced. A journal was kept after each interview to reflect on the role as an interviewer, gaps in knowledge and thinking, and the data collected.

### Analysis

The analysis followed an abductive approach, shifting between theoretical concepts and the participants' descriptions and lived experiences. Based on the recommendations of Atkins et als' (63), the manifest content analysis of Granheim and Lundmans (64) was chosen, due to the fact that evaluation often focuses on specific trial-related questions. The interviews were read through several times to get a sense of the whole. Texts that could provide an answer to the research questions were divided into meaning units which further was labelled with codes. The various codes were compared in terms of differences and similarities and sorted into categories. The process involved moving back-and-forth between the whole and parts of the text. The preliminary categories, in which content with commonalities was collected, and potential themes were discussed by all authors.

## Results

Several barriers and facilitators to ACP implementation in acute geriatric units were identified at three levels: clinical, organizational, and national. Additionally, participants described their experiences with how the ISP and their participation in the cRCT both facilitated and hindered the use of ACP. The findings below are presented according to these four main categories (see Table 4). Through the analytical process, it became evident that drawing clear distinctions between factors solely as barriers or as facilitators was not meaningful. For instance, some factors were perceived as barriers by some participants while others were neutral or perceived these factors as facilitators. Moreover, many factors were described as barriers when absent and consequently facilitators when present. This will be described in more detail below.

**Table 4:** Overview of barriers and facilitators.

The organizational level Hospital as context Resources and infrastructure Focus on treatment Management support The national level Challenges in management and prioritization of healthcare Coordination, documentation and communication between healthcare levels Technological solutions Incentives in favor of ACP The individual level Interest in ACP and motivation for implementation Personal barriers, personal suitability and knowledge The implementation support program and participation in the cRCT Implementation teams and dedicated roles in implementation effort Education and training, whole ward approach and train the trainer-model Integrating ACP into systems and local adaptations Resource persons and interdisciplinarity in implementation Research as both a barrier and facilitator Experiencing the benefits

### The organizational level

#### Hospital as context

There were different opinions on whether the acute geriatric units provided an adequate timing and context for ACP. Many of the participants argued that most patients admitted acutely to geriatric units were not suitable for ACP due to severe illness, delirium or cognitive impairment. This is further compounded by the short hospital stays, the frequent patient reassignments and the limited time to get to know the patient. Some were concerned that patients' wishes and values may be different in acute setting than in a more habitual state. There were also concerns around the preferences set in specialist healthcare may carry disproportionate weight when the patient is transferred to primary care. Some argued that it would be more effective if ACP were already initiated in primary care before admission to hospital.

[…] our experience has been that in our acute geriatric unit, although I don't have the statistics, there are quite a few patients for whom there will be limited value [to do ACP], where it's too late in the trajectory of their condition. (participant 7.1).

There is always a dilemma about when to have this conversation and who should do it, and everyone thinks someone else should do it. (Participant 7.1)

However, some believed acute geriatric units were well suited to ACP because the HCPs are trained to see the whole picture. One participant emphasized that patients are often not ready to talk about the topics in ACP until they are seriously ill:

They [physicians who are not motivated for ACP] don't feel that it's right to have these discussions if patients are not well enough. But there's a dilemma here, and I have thought a bit more about it [since starting the project]. It's when you're ill that you're most willing to talk about these things. Then, when you get better and you go home, you're less inclined to talk about it. […] So maybe it's not the wrong time [in acute geriatric units], it's the resources [lack of them]… (Participant 1.1).

To facilitate ACP implementation, some emphasized the need for simplified versions of ACP in the hospital context and suggested spreading ACP over several days or initiating it at the hospital and then continuing ACP in primary care or in an outpatient clinic at the hospital after discharge.

#### Resources and infrastructure

Resources and infrastructure were cited as both a major barrier to ACP implementation and a potential facilitator for better integration of ACP in acute geriatric units. The resource factor was described from different perspectives. Lack of resources was described as an existing barrier in the hospital context for both implementation effort and ACP. High workload, constant distractions, sick patients, overcrowding, shift work, deprioritization of professional development in favor of clinical activities and sick leave coverage, prioritization of acute needs, insufficient time, and lack of HCPs prevented prioritization of both ACP and implementation efforts:

We don't really have tasks that we can put aside to do that [ACP]. […] If we are to routinely offer that kind of structured conversation to the majority of our hospitalized patients, then it is… we don't have the resources to do that today, so we would have to reprioritize somehow, and I don't quite see a solution to that. (Participant 7.1).

[…] we are so caught up in the hustle and busyness of clinical tasks and organizational operations that the only one who manages to do a little [implementation effort] is me [the manager], who has perhaps the most flexible schedule. (Participant 3.1).

The clinical intervention outlined in this project was described by some participants as too comprehensive and time-consuming. Some described it as a logistical challenge to bring all parties together during a short and busy hospitalization, especially in relation to the relatives' geographical distance, work commitments or their own poor health. In addition, some HCPs were initially concerned that ACP could lead to unrealistic expectations from patients and their relatives or to requests that could not be met due to resource constraints, priorities or medical reasons (e.g., the harms outweigh the benefits). However, these concerns were no longer expressed as the implementation process progressed. Participants believed that increased staffing is essential, but some also argued that ACP is not necessarily time consuming:

But I think it's much more important to take the time to do something, because […] an ACP conversation doesn't have to take that much time, right? It's about having an extra conversation on one of the days during the hospitalization, isn't it? You only need to take half an hour, and you can achieve a lot in that half hour. (Participant 5.2).

The lack of suitable conversation rooms raised ethical and professional concerns, e.g., regarding confidentiality when another patient was present in a two-bed room. Another important logistical barrier was the lack of common meeting points for the different types of HCPs, both in implementation effort and when conducting ACP.

#### Management support

Almost all acute geriatric units stated that they would like more management support in the implementation of ACP, including frequent reminders to do ACP. It was strongly emphasized that the role of the manager is crucial in implementation effort:

I think it depends on the manager being fully committed to making this a priority. (Participant 6.1)

Participants argued that effective management support requires managers to believe in the intervention, be committed to its implementation, and motivate clinicians. The managers themselves noted that they need interest and commitment at the clinical level to actively engage in the process. In addition, some participants suggested that shared leadership between nurses and physicians, rather than the current split, would better facilitate the whole ward implementation. While participants appreciated the reminders from the research group to continue their implementation effort, some expressed a desire for increased support from the research group leadership directed towards their managers, serving as friendly reminders to enhance ACP implementation.

I believe that constant and friendly reminders have been absolutely necessary for us to be able to carry out and maintain it. (Participant 3.1).

#### Focus on treatment

Participants noted that treatment-oriented medicine in hospitals can be a barrier to ACP implementation. Some called for a culture change, pointing out that the focus is often on treatment options and limitations rather than value-based issues. Some spoke of a high threshold to talk about death, both for HCPs and for society as a whole:

We are traditionally much more focused on the treatment level and treatment limitations […]. […] if we are going to have a conversation that involves more value-oriented questions […], I think we easily slip into more practical oriented issues… whether they want treatment, whether we should set limits on treatment… It's more natural for us to focus on treatment… so that's a barrier in terms of having a more structured approach to having a comprehensive ACP conversation. (Participant 7.1)

Yes, perhaps there is also a need for a shift in culture out there too, and discussion of these issues may feel distant. It's not just within the biomedical model, but death has been removed from homes in our society. (Participant 3.2).

Others believed that the existing and underlying culture in acute geriatric units would value ACP. Some suggested that ACP could reinforce and be reinforced by the ongoing focus on avoiding excessive treatment in Norwegian hospitals and healthcare policies.

### The national level

#### Challenges in management and prioritization of healthcare

Some participants cited health management and prioritizations as barriers to ACP implementation. Some experienced that patients who would benefit most from geriatric care were often not admitted to these specialized units, which meant that patients best suited for ACP were admitted to other medical units. Additionally, some argued that there are geographical variations in geriatric care that could further hinder the provision of ACP more widely. Some participants discussed the high “production pressure” in hospitals, which leads to poor prioritization and inefficiency, ultimately sidelining patient communication. Participants acknowledged that solutions to these structural barriers appear to be quite complex but called for greater national prioritization of geriatrics and better alignment between strategic plans and local implementation, emphasizing the need for top-level directives to drive meaningful change.

#### Coordination, documentation and communication between healthcare levels

Some participants mentioned the lack of coordination, documentation and communication systems between different levels of the healthcare system as a barrier to ACP implementation. They emphasized that without accessible, structured documentation of ACP that can follow the patient throughout the healthcare system, conducting ACP would be pointless. They argued that proper documentation of ACP would ensure continuity of care and facilitate the transfer of important information and decisions about the patient.

#### Technological solutions

Some suggested modernizing with digital solutions, such as equipping all patient rooms with screens to enable remote participation by relatives, overcoming geographical, logistical and other barriers to ACP involvement. This facilitator would also serve purposes beyond ACP, such as facilitating contact with relatives more generally.

#### Incentives in favor of ACP

Several participants emphasized that financial incentives would promote the use of ACP:

A reimbursement rate would certainly help, because then management would give it more thought. (Participant 1.1).

Codes and reimbursement rates, I think they are… they're probably the primary “drivers” or incentives when it comes to having something implemented… like a “checklist”, right? […] So I believe that having these in place would make us better at remembering [ACP][…]. (Participant 5.1).

Others felt that financial incentives would not be enough to outweigh their belief that the lack of time and resources in acute geriatric units was a major barrier. One manager emphasized the need for specific directives at a higher level in the hospital organization to explicitly work on ACP. Others suggested including ACP in quality indicators and deviation reporting systems as incentives to promote its implementation.

### The individual level

#### Interest in ACP and motivation for implementation

Participants, whether regular clinicians or members of implementation teams, struggled to distinguish between a possible difference in interest in ACP and motivation to engage in implementation effort. They reported varying levels of interest in ACP among their colleagues: “There are doctors who are not very interested” (participant 8.1). Participants believed that although nurses were interested in ACP, there was little motivation to implement it. Low motivation was possibly due to a lack of understanding and/or scattered information about the project, delays between ACP training and when units began to conduct ACP, and a lack of time for professional development and continued training in ACP. However, some believed that the main barrier was a lack of time rather than lack of interest or motivation.

#### Personal barriers, personal suitability and knowledge

Although standardized ACP was not a previously routine practice in these acute geriatric units, physicians felt confident in their ability to have ACP conversations. While few physicians cited a lack of knowledge, some nurses expressed this concern. Some noted that not all HCPs are suited to having these conversations and that talking about death can be challenging for some.

And I think there are personality barriers. I mean, who is the physician? I think that many can do a good job […] but I don't think everyone can get to that point […]. (Participant 3.1).

### The implementation support program and participation in the cRCT

Overall, the participants reported different experiences regarding their exposure to the ISP, both within and between units. Implementation team members were generally more familiar with the ISP, while regular clinicians tended to have less experience. Additionally, some units received more training and implementation support than others, as they made greater use of the resources offered by the research group, which will be described elsewhere. Not all acute geriatric units tested all the recommended implementation strategies and measures outlined in our ISP (please see Table 1 for the ISP).

#### Implementation teams and dedicated roles in implementation effort

The acute geriatric units faced challenges in establishing and maintaining implementation teams due to scheduling conflicts, clinical workload, staff turnover, sick leaves, and recruitment issues. Few implementation teams had dedicated time for implementation effort. Most units struggled to follow up the recommended action plan for ACP implementation. Furthermore, some participants struggled to understand their role within the implementation team and the process. However, one unit successfully maintained a stable implementation team with clearly defined roles and a dedicated coordinator who oversaw implementation, kept track of potential available patients, and encouraged physicians to engage in ACP. Additionally, the nurses on this implementation team had some dedicated time for professional development and implementation effort. This implementation team emphasized the importance of systematically distributing responsibilities:

It's nice to have some people who we know can integrate things into the system. I for one, I know that it's very reassuring to be able to talk to those in the implementation team and that things will get done when we… that we are several people involved together. (Participant 2.2).

It has been crucial [to have a coordinator to oversee implementation and ACP]. (Participant 2.1).

To facilitate ACP implementation, some participants suggested appointing an ACP coordinator at the organizational level of hospitals. This coordinator would be responsible for logistics and for conducting ACP with all eligible patients.

#### Education and training, whole ward approach and train the trainer-model

Education, training, supervision, and support were offered free of charge and through a train-the-trainer model and a whole ward approach. Participants reported mixed experiences regarding the educational program's usefulness. HCPs in the implementation teams found the on-site teaching and the ACP simulation facilitated by the research group to be beneficial. The implementation teams were invited to a full-day ACP simulation led by a phycologist, which was well received. However, during the simulations with regular clinicians in local context, some physicians expressed discomfort and resistance, while most units struggled to engage nursing staff.

There were differences in who received which aspects of the ACP training program, particularly among regular clinicians outside the implementation teams. The acute geriatric units faced the challenge of achieving a whole ward approach and maintaining continuous training through the train-the-trainer model. ACP was often mainly conducted by members of the implementation teams. The main barriers to reaching regular clinicians included limited time for professional development, lack of common meeting points for physicians and nurses, high staff turnover and sick leaves, physicians' low interest in prioritizing ACP, and limited involvement of nurses in the project and their low capacity for additional clinical tasks.

[…] so it is very difficult for us in our clinical day-to-day basis to find time for that training. We would need external help for this. (Participant 7.1).

Some geriatricians described “learning by doing” as the most effective training approach for them. To enhance engagement with the other clinicians in the units, participants suggested several solutions: Regularly addressing ACP in meetings, coordinated training efforts between all HCPs, providing timely access to ACP materials, and offering concise training sessions on 2–4 specific dates to increase attendance. There were differing opinions on whether training should be provided by external or local experts.

##### ACP materials and resources

The research group developed various tools to facilitate ACP implementation in collaboration with the intervention units. However, not all resources, including materials on barriers and possible solutions, as well as ACP educational videos, were fully utilized. Reported reasons for this included late introduction, inadequate distribution and communication within the local setting, and a lack of time and structure for local education.

Many physicians argued that the pocket card and documentation template, which can be integrated into the electronic patient journal system, were sufficient for ACP education. An educational video demonstrating ACP was also seen as a valuable resource. While few participants had distributed the information leaflets to patients and relatives, those who did found them helpful in preparing patients for the ACP conversation.

##### Dialogue and network meetings for implementation teams

Participants valued the regular digital dialogue meetings and the annual in-person network meetings, where the research group and intervention units discussed current challenges and experiences. However, attendance was relatively low, with typically only 2–3 out of 5–6 units represented. Reported reasons were lack of time and inability to step away from clinical responsibilities. Participating HCPs emphasized that these meetings fostered engagement by maintaining communication with the research group, enabling their involvement in developing the clinical intervention, and facilitating the collective sharing of experiences and challenges:

And those gatherings… there was so much motivation and momentum in these meetings, which has helped us to keep going. (Participant 3.1).

#### Integrating ACP into systems and local adaptations

Some participants emphasized the importance of integrating ACP into systems:

[…] if we could somehow establish this in a systematic way, I believe that overall, in the healthcare system, we could achieve time savings, right? We might be able to provide more tailored healthcare services. And we could perhaps even offer more appropriate treatment. […] if we had a system for that, we could avoid some very tragic patient trajectories that are very burdensome for patients and their relatives, but also for us healthcare professionals who spend a lot of resources. (Participant 7.1).

However, participants emphasized the importance of local adaptations and flexible standardization of ACP during implementation.

#### Resource persons and interdisciplinarity in implementation

Participants highlighted the need for resource persons or “champions” to drive implementation and motivate other clinicians. Furthermore, interdisciplinarity and role allocation were seen as important in both implementation and conducting ACP. In this project, physicians primarily led ACP conversations, with occasional involvement from nurses. While physicians felt responsible for clarifying prognoses and treatment options, they acknowledged that particularly experienced nurses could manage aspects of ACP. Nurses valued ACP but cited time constraints and lack of knowledge as significant barriers. However, both physicians and nurses expressed a desire for greater nurse involvement in ACP implementation. Some suggested dedicating time for professional development would enhance nurses' participation in both ACP conversations and its implementation, including the identification of eligible patients and leading the conversations alongside physicians:

… and I think the nurses… they talk a lot with… they spend a lot of time with the patients. So I think they have a unique opportunity to gather a lot of information from the patients themselves in terms of their hopes and wishes, and… Yes… I mean, not necessarily about prognosis or the way forward, but more about these overarching issues. (Participant 4.1).

#### Research as both a barrier and a facilitator

The research group particularly engaged the acute geriatric units in developing ACP materials, which some participants emphasized as crucial, arguing that sustainable implementation must originate at the clinical level. While many appreciated the project's attentiveness to local needs during implementation, others felt that the action research design hindered progress and local implementation, which ultimately affected motivation.

During the implementation phase, HCPs primarily focused on including patients and relatives to the questionnaires associated with the cRCT rather than on the ISP. Some participants struggled to understand the implementation science terminology, and many indicated that their involvement in the cRCT required considerable additional work, time, and concern:

I find it a bit difficult now when you [the researcher asks about experiences with implementation] mention implementation, because right now… at the moment, we are focusing on inclusion [of patients and relatives to the cRCT part of the project], right? I think as long as we are including [for the cRCT], that's what we're focusing on. (Participant 2.1).

Yes, there's no room for both. (Participant 2.4).

However, some participants emphasized that the research aspect also acted as a catalyst for gaining experience with ACP and its benefits. Others found it motivating to help explore the appropriate context for ACP implementation:

[…] I think if we can find out through this research project what role ACP plays in avoiding unwanted and burdensome overtreatment, and that we can contribute to evidence-based knowledge, then that is very motivating for me […]. (Participant 2.1).

#### Experiencing the benefits

Participants emphasized that experiencing the benefits of ACP creates a self-reinforcing cycle that generates further interest in ACP among other clinicians. The gratitude expressed by patients and their relatives highlighted the significance of ACP for HCPs:

I believe that a facilitator is to just do it and experience its benefits, and in order for it to be successful you must get as many people as possible to try it. (Participant 5.1).

I believe that a facilitator is to look at patients who do not want more treatment, where relatives are pushing for it [and an ACP conversation has taken place]. I think that might be the best outcome of this study. (Participant 8.1).

However, some participants noted that few patients currently have documented ACPs, which limits the perceived overall benefits of the intervention. One participant argued that the goal should be to make ACP more widespread and therefore more valuable.

Several participants argued that geriatricians already integrate elements of ACP into their routine practice. However, some noted that these conversations often focus primarily on treatment levels. In contrast, discussions about patients' preferences for information, their desired level of involvement in decision-making, the role of relatives, and discussions related to values, hopes, and concerns are reported as newer aspects for some participants:

“What do you think is the most important thing for you in your daily life […]?” Because often we just run around and get on with it without considering what the patient actually wants, and that could perhaps lead to the biggest changes. (participant 2.3).

## Discussion

This study explored the barriers, facilitators, and experiences related to ACP implementation among HCPs in acute geriatric hospital units. Regular clinicians reported less experience with the ISP, partly because certain aspects of the ISP were targeted at the implementation teams, and partly due to limited exposure to the ISP for those outside these teams. This lack of exposure can be linked to barriers in the first three categories: The organizational, national and individual levels. Below, we discuss some of the challenges faced in ACP implementation and offer suggestions to address these barriers.

While implementation strategies and measures are essential for adopting evidence-based practices and ensuring sustainability (65), the barriers identified in this study—some of which were already present in this clinical context—may have been so significant that the strategies and measures intended to facilitate ACP implementation proved insufficient. A recurring barrier in this study was the overwhelming workload in these acute geriatric units. Lack of time and resources was the main barrier to both clinical intervention and implementation effort, which is also cited in other studies (3, 66). This highlights a critical paradox: lack of time and resources to implement interventions that could ultimately save resources. This paradox leads to a self-reinforcing cycle in which immediate patient care consumes resources without addressing the underlying inefficiencies. Furthermore, this study revealed reciprocal effects between barriers to the clinical intervention and implementation strategies and measures. Lack of time not only hindered doing ACP conversations, but also the implementation effort, when trying to implement something that was challenging to carry out. Ignoring either side of this equation creates a “hamster wheel” effect, where implementation stalls due to perceived clinical impracticality, while clinical engagement remains limited due to insufficient national and organizational support and a lack of experience with the benefits of ACP. Additionally, the research component of this project, the cRCT, required some effort, which often overshadowed implementation effort in the acute geriatric units. This highlights the tension between generating evidence and achieving effective implementation. Both this study and another substudy (62) found that competency in implementation science within healthcare is low, including the awareness of systematic integration of implementation effort and their evaluations.

Overall, the acute geriatric units struggled to implement ACP, with some units struggling more than others. Some of the reasons for this can be explained by their size and the local resources available. Those that had less difficulty either had a stable, interdisciplinary implementation team with a dedicated ACP coordinator and allocated time for professional development for nursing staff, or a dedicated manager who took on extensive responsibilities for implementation. Furthermore, the projects' intention to adopt a whole ward (59) and a train-the-trainer approach (60) faced significant challenges. It was a challenge to effectively reach all HCPs with training, especially nursing staff, and to integrate both annual training and the training of new employees into the system. Establishing interdisciplinary collaboration and finding common meeting points between the different HCPs seems to be an important infrastructural facilitator to both ACP conversations and the implementation of complex interventions. Notably, the potential for involving nurses in both ACP conversations and implementation effort may have been underutilized in this project, despite their essential role in the discussion, planning, execution, and evaluation of interventions (67).

Nurses reported a lack of knowledge regarding ACP, while geriatricians felt competent and prepared to engage in ACP, viewing it as aligned with the values of geriatric care. However, this did not automatically translate into more ACP conversations. Research indicates that clinical priorities are often dominated by traditional biomedical approaches, resulting in under-provision of psychosocial needs, comprehensive care, and clinical communication (68, 69). In addition, the participants expressed concern about the “production pressure” in healthcare, where cost reduction and increased efficiency have been the driving force in recent decades (70). This biomedical and production-oriented system, coupled with a lack of clear incentives in favor of ACP, makes it difficult to prioritize clinical communication. A review on quality of care at the end of life highlighted that failure to discuss care with patients and their relatives can lead to clinical uncertainty and unhelpful aggressive treatment that ultimately leads to distress (71). Without management support, explicit recognition of the long-term benefits of ACP or incentives for its adoption, it may be difficult for clinicians to justify the reallocation of time and resources to its implementation. Given the current realities of the healthcare system, clinicians could benefit from support in recognizing the value of evidence-based interventions to facilitate their adoption. This creates a tension in which ACP is recognized as important but is overshadowed by more immediate, task-oriented medical responsibilities. This situation may be highly problematic in a society where over- and under treatment is rife.

A review of systematic reviews of ACP emphasizes the importance of adopting a whole-system strategic approach. This perspective views ACP as an interconnected network of interdependent components. Establishing a structural foundation from a system perspective is essential. This includes legislation and policy structures that have a positive impact on healthcare institutions and promote social and cultural awareness of ACP (43). Kalager (72) argues that the health system itself, rather than research funding, should ensure necessary implementation of national recommendations. Educational initiatives and investment in the implementation of complex interventions, which may demand upfront costs but are likely to provide long-term benefits, can create a more sustainable and efficient health system.

## Strengths and limitations

By integrating continuous participant feedback (63) and qualitative data collection, this study provides a comprehensive understanding of the factors influencing implementation in a relatively unexplored context. However, the pre-existing barriers in this context limit our ability to assess whether the implementation strategies and measures would have been more effective under different conditions or whether problems with the clinical intervention, study design, or the strategies and measures themselves occurred. Another strength of this study is the reporting of the implementation strategies and measures used. To our knowledge, the CFIR is not commonly used in the planning, reporting or evaluation of ACP. The CFIR-inspired interview guide facilitated exploration of different domains and offered a broader view of barriers and facilitators. Another potential limitations include the involvement of researchers in both the ISP and the evaluation and data collection. Additionally, at the time of the semi-structured interviews, participants had only attended a few structured ACP conversations. Those interviewed may have had a particular interest in ACP or related topics.

## Conclusion

This study identified significant barriers and facilitators to ACP implementation in acute geriatric hospital units at individual, organizational and national levels. Clear recommendations for successful ACP implementation remain elusive, partly due to the pre-existing barriers in this context. Participants reported lack of time and resources as the main barrier to ACP implementation, revealing a paradox: There is no time and resources to implement an intervention that could potentially free up resources in a larger perspective. To address these challenges, a whole-system approach that integrates policy, external incentives, educational initiatives and strategic support as well as investments in implementing complex interventions that offer long-term benefits despite initial costs, seems necessary.

## Data Availability

The data presented in this article are not available due to privacy concerns. Requests to access the data should be directed to l.b.broderud@medisin.uio.no.
