# Supplementary material for: Barriers and facilitators in implementing advance care planning for frail older patients acutely admitted to geriatric hospital units: a nested qualitative study
Source: Front Health Serv. 2025 Dec 17;5:1646541. doi: 10.3389/frhs.2025.1646541 (PMC12753930; doi:10.3389/frhs.2025.1646541)
Supplement: Supplementary file 1 [file Table1.docx]

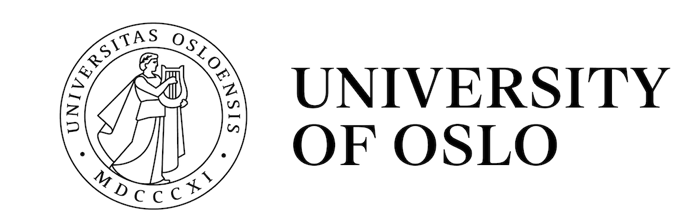


*Implementation of advance care planning in the routine care of acutely admitted patients in geriatric units: A cluster-randomized controlled trial*

INTERVIEW GUIDE

## Introduction

This interview is part of the ongoing study "Implementation of Advance Care Planning in Acute Geriatric Units – a Cluster Randomized Study." The aim of the project is to improve healthcare services, user participation, and quality of life for severely ill, home-dwelling elderly individuals and their relatives in an effective, sustainable, and coordinated manner through better implementation of Advance Care Planning (ACP). ACP is now recommended in the Norwegian healthcare system. In this project, we aim to implement a training program for conducting ACP in acute geriatric hospital units and to evaluate the implementation systematically.

ACP has been implemented to a limited extent in a systematic way within the Norwegian healthcare system, despite recommendations both nationally and internationally (References: Nasjonal veileder: Beslutningsprosesser ved begrensning av livsforlengende behandling (2013), Meld. St. 24 (2019-2020) Lindrende behandling og omsorg, NOU 2017/16 På liv og død). We would like to hear about your experiences regarding the implementation and use of ACP so far, with a particular focus on what may facilitate or hinder ACP.

*Introducing the interviewers. Allow the participant to introduce themselves with their name and profession.*

*The framework for the interview: It’s helpful to use concrete examples, but please avoid giving information that could lead to the identification of the cases you may discuss, to ensure confidentiality is maintained. The interview will last 1 to 1.5 hours and will be recorded and transcribed afterward. Everything you say will be treated confidentially and anonymized. Participation is voluntary, and you may withdraw at any time without explanation.*

*Start the audio recording.*

## Questions

Opening: We would like to hear about your positive and negative experiences, both with the implementation and with the ACP itself. In this way, we can learn from you and improve our work. By “implementation” we mean the process of getting ACP into routine clinical practice within the unit.

1. **Experiences with the implementation support from the project group and local implementation efforts** (Ask about experiences regarding implementation teams, ACP coordinator, training, supervision, learning resources and ACP materials, e.g., guidelines, pocket cards etc.)

- What kind of implementation support have you received? (By 'implementation support,' we mean various measures and support from the project group and the implementation team to introduce and conduct ACP)
  - How have you experienced the implementation support? (Refer to the points in the heading)
  - Suggestions for improvements/changes?
- What are the most important changes that have occurred in the unit since the project started? What actions have you taken locally? What worked/did not work?

1. **Barriers and facilitators related to ACP implementation**Now that you have been working more systematically with ACP...

a) What have you experienced as the most important barriers to conducting ACP? (By barriers, we mean factors that can hinder ACP; this can also include the absence of facilitators - see below. Has anything changed in this regard during the project period?)

- At the clinical level: Lack of knowledge, training, supervision, personal experience; challenges regarding self-efficacy, documentation, timing, assessment of consent capacity.
- At the organizational level (unit/hospital): Lack of time, resources, physical conditions (suitable rooms), local adaptations for implementation, systems/routines, management engagement/support, challenges related to culture and attitudes (death as a taboo/biomedical model), prioritization, high turnover. (If time/resources are mentioned, ask if they would conduct more ACP conversations if they had more time.)
- At the national level: Lack of collaboration between healthcare levels (common documentation and communication systems), national guidelines, national initiatives and prioritizations, incentives (tariffs, mandates, assignments, quality measurement), education, legislation.
- How would you describe your motivation regarding ACP implementation? If low motivation, why?
- Is there a difference in motivation for the ACP conversations themselves versus implementing it?
- Is everyone in the ward “on board”? Is there a shared understanding of why and how you work with ACP in the ward? Role distribution: What role and responsibility do you believe you as healthcare professionals have in conducting these conversations? Are there variations related to professional background?

b) What have you experienced as facilitators/success factors for implementing ACP in your ward? (By facilitators, we mean factors that can promote the adoption and implementation of ACP; this can also include the absence of barriers or the better management of those barriers (see above)). Has any of this changed during the project period?

- At the clinical level: Training and guidance, ethical reflection/reflection groups, demonstrating benefits/raising awareness within the professional community, learning resources/ ACP materials.
- At the organizational level: Establishing systems and routines, prioritization/clear directives, knowledge of benefits/effect of ACP, manager support, cultural/attitude change, local adaptations for implementation.
- At the national level: Collaboration (shared communication systems), national guidelines, incentives (fees, mandates in laws or guidelines, assignment letters, quality measurements), education, public awareness, explicit legislation for ACP.
- Do you have experience with initiatives/facilitators that have helped to overcome barriers?
- What could have been done differently (facilitators) regarding making ACP work in your organization?

1. **At the end:** Is there anything else we should be aware of that we haven't asked about?
